# Appraising ascorbic acid as a chemoprevention agent for acute myeloid leukaemia using Mendelian Randomisation

**DOI:** 10.1038/s41408-024-01168-7

**Published:** 2024-10-18

**Authors:** Sina A. Beer, Molly Went, Jessica M. Hislop, Richard Houlston, Martin Kaiser

**Affiliations:** 1 Division of Genetics and Epidemiology, The Institute of Cancer Research, Sutton, Surrey SM2 5NG UK; 2 The Royal Marsden Hospital NHS Foundation Trust, London, UK

**Keywords:** Cancer epigenetics, Acute myeloid leukaemia

Although acute myeloid leukaemia (AML) is one of the most common haematological malignancies in adults, its aetiological basis is poorly understood [1]. The only well recognised modifiable risk factors are smoking and exposure to benzene [2]. There is increasing evidence for the redox system playing a significant role in both the development and progression of AML [3]. *TET2* mutations in haematopoietic stem and progenitor cells (HSPCs) can disrupt this system, leading to uncontrolled proliferation of malignant cells. These mutations are among the most frequent cancer-driving lesions contributing to AML oncogenesis [4]. *TET2* functions as an ascorbic acid (AA)-dependent dioxygenase, and treatment with AA has demonstrated two effects, mimicking *TET2* restoration [5], and inducing apoptosis in AML cells [6]. In addition to preclinical studies and case reports [5, 7], recent clinical trial data further supports the therapeutic value of AA in slowing disease progression [8, 9]. Since *TET2* mutations occur in the early stages of preleukemic clone formation and are a feature of pre-malignant age-related clonal haematopoiesis of indeterminate potential (CHIP) [10], AA may represent an attractive chemotherapeutic agent a priori.

Although randomized controlled trials (RCTs) are the gold standard for evaluating the effectiveness of interventions, they are often impractical due to costs, time constraints, or ethical issues. One alternative is to perform Mendelian randomization (MR), which is analogous to RCTs in study design. The MR method uses genetic variants related to modifiable exposures as tools to detect causal associations with outcomes. Since genetic variants used as instrumental variables (IVs) are randomly allocated at conception, MR studies are less prone to confounding and reverse causality. To explore the potential of AA supplementation as a chemoprevention agent for AML, we investigated the relationship between circulating AA levels and the risk of AML and CHIP using a two-sample MR (2S-MR) framework (Fig. 1). Single nucleotide polymorphisms (SNPs) associated with AA levels were identified from genome-wide association studies (GWAS). We then assessed the association of the IV with AML and CHIP in large GWAS datasets. Ethical approval was not required for this project as all the data came from summary statistics of published GWAS, and no individual-level data were used.

**Fig. 1:**
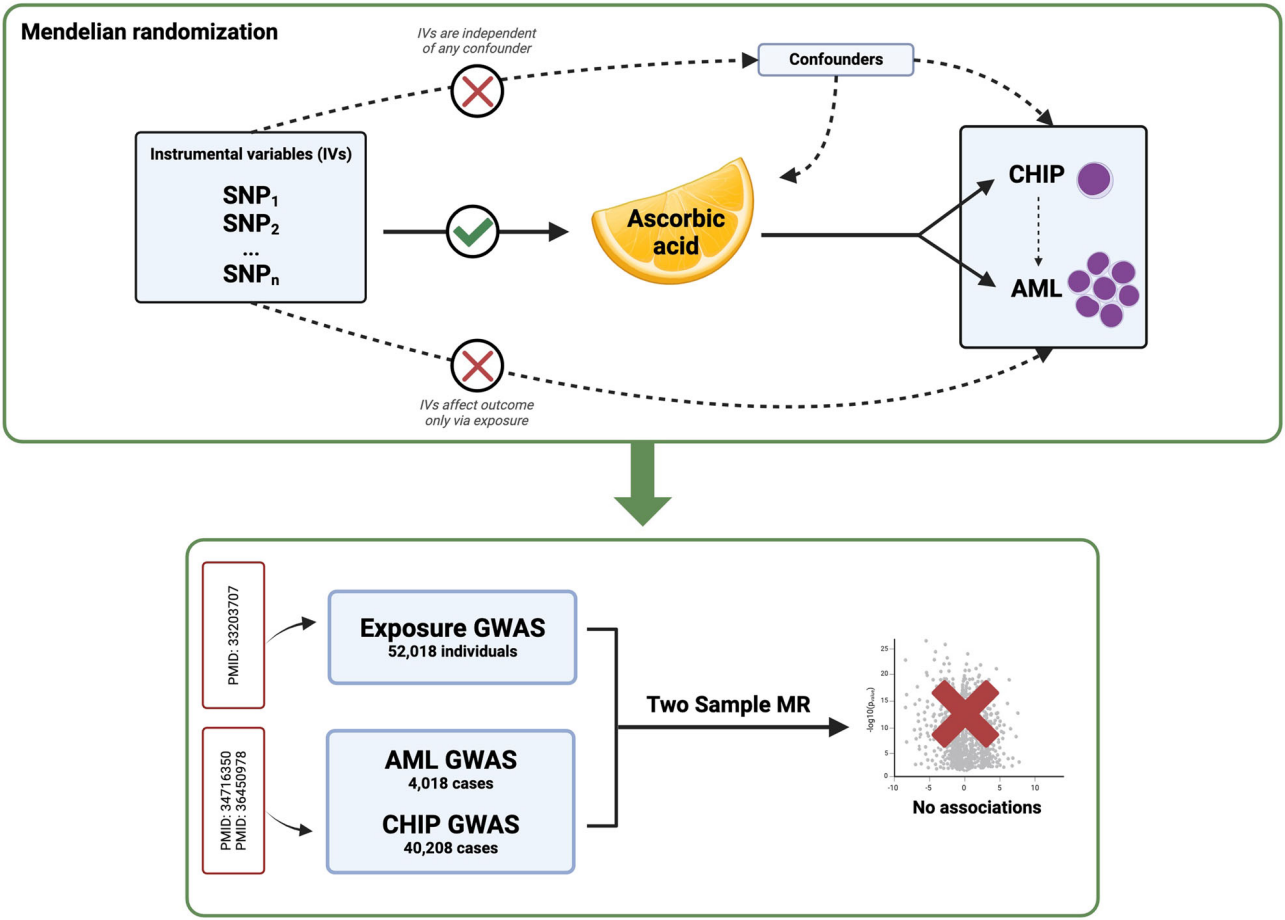
Mendelian randomisation (MR) study design and result summary. MR has three key prerequisites for using single nucleotide polymorphisms (SNPs) as instrumental variables (IV): (1) The IVs must be independent of any confounders, (2) the IVs must be associated with the exposure, (3) the IVs should influence the outcome only through the exposure. (Upper part of diagram) Illustration of the MR approach using SNPs associated with the exposure ascorbic acid (AA) and its influence on the development of clonal haematopoiesis of indeterminate potential (CHIP) or acute myeloid leukaemia (AML). (Lower part of diagram) Depicts a Two-Sample MR (2SMR) analysis, where summary statistics from large genome wide association studies (GWAS) datasets were used to investigate a causal relationship between AA and CHIP/AML. No significant association was found. Created with BioRender.com.

We performed a 2S-MR using the TwoSampleMR package [11], utilizing meta-analyses of 4018 AML cases [12] and 40,208 CHIP cases, including 3918 cases with *TET2* mutations [13]. After filtering, we identified 16 independent SNPs for AML and 18 SNPs for CHIP (r² < 0.01, minor allele frequency (MAF) > 0.01) associated with AA levels (*P* < 1 × 10^–6^) in 52,018 individuals of European ancestry. We harmonised genetic variants to ensure that the effect estimates on AA and AML/CHIP risk referred to the same allele (Supplementary Data 1). If the sentinel SNP was not present in the outcome dataset, we used highly correlated proxy SNPs (i.e. r^2^ > 0.8). The estimated proportion of the variance explained (PVE) was calculated using lifetime risk estimates for AML of 0.5% [13] and CHIP of 10% [14]. For the primary analysis we used the Wald-ratio and random-effects inverse variance weighted (IVW-RE) method. We considered weighted median, weighted mode, and MR-Egger in sensitivity analyses, since these methods make different assumptions regarding pleiotropy and outliers (Supplementary Data 2). Analyses were conducted using R v4.2.1. A *P*-value < 0.05 (two-sided) was considered statistically significant.

Genetically predicted circulating levels of AA were not associated with the risk of developing AML (odds radio per standard deviation [OR_SD_] = 1.35, 95% confidence intervals [CI]: 0.94–1.94, *P*_*IVW-FE*_ = 0.104, Table 1). There was also no support from the 2SMR analysis for a relationship between genetically predicted AA levels and the risk of all CHIP (OR_SD_ = 0.99, 95% CI: 0.89–1.12, *P*_*IVW-RE*_ = 0.98 and *P*_*IVW-FE*_ = 0.97, Table 1). To explore the possibility that the impact of AA might be confined to *TET2*-mutated CHIP, we undertook a subtype analysis on the 3918 *TET2*-mutated CHIP cases. However, restricting data analysis to this subtype provided no evidence for a relationship between genetically predicted AA levels and a risk of developing *TET2*-mutated CHIP (OR_SD_ = 0.93, 95% CI: 0.70–1.21, *P*_*IVW-RE*_ = 0.58, Table 1).

**Table 1:** Results of two-sample mendelian randomization analysis to infer the causal relationship between ascorbic acid (AA) and AML/CHIP.

Outcome	Method	Number of SNPs	p-Value	Odds Ratio	Lower 95% CI	Upper 95% CI
AML	IVW-RE	15	1.04E-01	1.35	0.94	1.94
AML	IVW-FE	15	1.04E-01	1.35	0.94	1.94
AML	ML	15	9.68E-02	1.36	0.95	1.96
AML	SM	15	2.57E-01	1.36	0.8	2.3
AML	WM	15	1.96E-01	1.41	0.84	2.36
AML	SMd	15	1.24E-01	2.07	0.87	4.94
AML	WMd	15	1.55E-01	1.68	0.85	3.32
AML	MR-Egger	15	2.79E-01	1.81	0.65	5.08
CHIP (all)	IVW-RE	17	9.80E-01	1.0	0.89	1.12
CHIP (all)	IVW-FE	17	9.74E-01	1.0	0.91	1.09
CHIP (all)	ML	17	9.75E-01	1.0	0.91	1.09
CHIP (all)	SM	17	3.15E-01	0.93	0.81	1.07
CHIP (all)	WM	17	6.68E-01	0.97	0.86	1.1
CHIP (all)	SMd	17	3.93E-01	0.92	0.76	1.11
CHIP (all)	WMd	17	6.95E-01	0.98	0.87	1.1
CHIP (all)	MR-Egger	17	9.54E-01	0.99	0.82	1.21
CHIP (TET2)	IVW-RE	17	5.81E-01	0.93	0.71	1.21
CHIP (TET2)	IVW-FE	17	4.92E-01	0.93	0.75	1.15
CHIP (TET2)	ML	17	4.90E-01	0.93	0.74	1.15
CHIP (TET2)	SM	17	3.98E-01	0.86	0.6	1.22
CHIP (TET2)	WM	17	6.77E-01	0.94	0.71	1.25
CHIP (TET2)	SMd	17	6.98E-01	0.91	0.55	1.48
CHIP (TET2)	WMd	17	7.36E-01	0.95	0.71	1.27
CHIP (TET2)	MR-Egger	17	7.40E-01	0.92	0.58	1.46

*IVW-RE* inverse variance weighted - random effects, *IVW-FE* inverse variance weighted - fixed effects, *ML* maximum likelihood, *SM* simple median, *WM* weighted median, *SMd* simple mode, *WMd* weighted mode.

In conclusion, our study does not support the use of AA supplementation as a chemoprevention agent for AML. While our study was well-powered, with over 80% power to detect a 33% risk reduction (Supplementary Data 2), we cannot rule out the possibility of a more marginal impact. Furthermore, our study was not configured to provide evidence of AA as a therapeutic agent in the context of established AML.

## Supplementary information

Supplementary Data 1

Supplementary Data 2

## Data Availability

The summary statistics for clonal haematopoiesis outcome data are available from the GWAS Catalog (https://www.ebi.ac.uk/gwas/publications/36450978). The summary statistics for acute myeloid leukaemia outcome data are available from the GWAS Catalog (https://www.ebi.ac.uk/gwas/publications/34716350). The summary statistics for vitamin C exposure date are available from the GWAS Catalog (https://www.ebi.ac.uk/gwas/publications/33203707). Analysis in this study is adapted from code available in our public repository: https://github.com/houlstonlab/MR-PheWAS. The datasets and analysis scripts are available from the corresponding author upon reasonable request.
