# Supplementary material for: Appraising ascorbic acid as a chemoprevention agent for acute myeloid leukaemia using Mendelian Randomisation
Source: Blood Cancer J. 2024 Oct 18;14(1):183. doi: 10.1038/s41408-024-01168-7 (PMC11489766; doi:10.1038/s41408-024-01168-7)
Supplement: Supplementary file 2 — Supplementary Data 2 [file 41408_2024_1168_MOESM2_ESM.pdf]

Sensitivity Tests of 2SMR analysis

outcome	exposure	method	nsnp	Low_SNP_Count	Pleiotropy	Heterogeneity	Leave-One-Out_IVW
AML	AA	IVW-RE	15	FALSE	FALSE	FALSE	FALSE
AML	AA	IVE-FE	15	FALSE	FALSE	FALSE	FALSE
AML	AA	Max. likelihood	15	FALSE	FALSE	FALSE	FALSE
AML	AA	Simple median	15	FALSE	FALSE	FALSE	FALSE
AML	AA	Weighted median	15	FALSE	FALSE	FALSE	FALSE
AML	AA	Simple mode	15	FALSE	FALSE	FALSE	FALSE
AML	AA	Weighted mode	15	FALSE	FALSE	FALSE	FALSE
AML	AA	MR Egger	15	FALSE	FALSE	FALSE	FALSE
CHIP (all)	AA	IVW-RE	17	FALSE	FALSE	TRUE	FALSE
CHIP (all)	AA	IVE-FE	17	FALSE	FALSE	TRUE	FALSE
CHIP (all)	AA	Max. likelihood	17	FALSE	FALSE	TRUE	FALSE
CHIP (all)	AA	Simple median	17	FALSE	FALSE	TRUE	FALSE
CHIP (all)	AA	Weighted median	17	FALSE	FALSE	TRUE	FALSE
CHIP (all)	AA	Simple mode	17	FALSE	FALSE	TRUE	FALSE
CHIP (all)	AA	Weighted mode	17	FALSE	FALSE	TRUE	FALSE
CHIP (all)	AA	MR Egger	17	FALSE	FALSE	TRUE	FALSE
CHIP (TET2)	AA	IVW-RE	17	FALSE	FALSE	FALSE	FALSE
CHIP (TET2)	AA	IVE-FE	17	FALSE	FALSE	FALSE	FALSE
CHIP (TET2)	AA	Max. likelihood	17	FALSE	FALSE	FALSE	FALSE
CHIP (TET2)	AA	Simple median	17	FALSE	FALSE	FALSE	FALSE
CHIP (TET2)	AA	Weighted median	17	FALSE	FALSE	FALSE	FALSE
CHIP (TET2)	AA	Simple mode	17	FALSE	FALSE	FALSE	FALSE
CHIP (TET2)	AA	Weighted mode	17	FALSE	FALSE	FALSE	FALSE
CHIP (TET2)	AA	MR Egger	17	FALSE	FALSE	FALSE	FALSE

Power Table of 2SMR analysis

Outcome	Exposure	n_snp	r2	Sample Size	F-Statistic	Power 1.05	Power 1.1	Power 1.25	Power 1.33	Power 1.5
AML	AA	16	0.01	52018	42.02	0.06	0.09	0.3	0.46	0.78
CHIP (all)	AA	18	0.02	52018	63.15	0.2	0.61	1	1	1
CHIP (TET2)	AA	18	0.02	52018	63.15	0.07	0.15	0.62	0.85	0.99
